# Disaster Preparedness and Response Among Healthcare Professionals During the Hajj: A Systematic Literature Review

**DOI:** 10.3390/healthcare13131571

**Published:** 2025-06-30

**Authors:** Thawab Alrabie, Michael Brown, Billiejoan Rice, Lynne Marsh

**Affiliations:** 1 School of Nursing and Midwifery, Queen’s University Belfast, Belfast BT9 7BL, UK; l.marsh@qub.ac.uk; 2 Nursing Practices, Nursing Collage, Umm Al Gura University, Makkah 24382, Saudi Arabia; m.j.brown@qub.ac.uk (M.B.); b.j.rice@qub.ac.uk (B.R.)

**Keywords:** disaster preparedness, emergency response, nurses’ disaster insights, disaster response, disaster nursing, emergency preparedness

## Abstract

**Background**: Disasters pose significant challenges to public health by disrupting essential services, especially during mass gatherings such as the Hajj pilgrimage. These complex events demand swifts coordinated action from healthcare professionals. However, many remain insufficiently equipped for large-scale emergencies due to gaps in triage training, disaster knowledge, and established response protocols—issues that are particularly critical in high-risk settings. **Aim**: This systematic literature review aims to explore the clinical insights of registered nurses and other healthcare professionals in disaster preparedness and response during the Hajj pilgrimage in Saudi Arabia. **Methods**: A systematic literature review was conducted following PRISMA guidelines. A comprehensive database search was performed across CINAHL, Scopus, Medline, Embase, and APA PsycINFO, focusing on studies published between 2012 and 2025. Eligible studies addressed disaster nursing education, preparedness, and response. The Mixed Methods Appraisal Tool (MMAT) was used for quality assessment. **Results**: Twenty-three studies met the inclusion criteria. Three main themes emerged: disaster preparedness, experiences and challenges in disaster response, and education and training. The review revealed lack in nurses’ readiness, policy awareness, and real-world disaster experience. Training and curriculum improvements are essential to enhance disaster response capabilities. **Conclusions**: This review highlights the urgent need for standardized disaster nursing curricula to enhance preparedness and response competencies. Incorporating structured disaster training into nursing education will strengthen healthcare system resilience. Future research should adopt qualitative methods to capture healthcare professionals lived experiences during the Hajj. Including diverse participants and focusing on the unique cultural context can enhance disaster preparedness and response strategies.

## 1. Introduction

Disasters are unforeseen events that disrupt essential services such as transportation, medical care, communication, water supply, and sanitation [1]. They pose significant threats to communities, leading to casualties, displacement, and infrastructural damage, often requiring coordinated responses from local, national, and international entities [2]. The World Health Organization (WHO) defines a disaster as a sudden ecological phenomenon that necessitates external assistance [3]. Disasters are broadly classified into natural disasters, such as floods, earthquakes, and hurricanes, and anthropogenic disasters, such as fires, mass population movements, transportation accidents, armed conflicts, and chemical spills [4].

Among these disasters, mass gatherings represent a unique and increasingly relevant subset of events that demand context-specific disaster preparedness strategies. One of the most prominent examples is the annual Hajj pilgrimage in Saudi Arabia, which draws over two million pilgrims from diverse cultural and linguistic backgrounds. The event takes place over several days in a geographically confined area, involving dense crowds, high physical exertion, extreme heat, and complex ritual movements. These conditions, combined with the heightened emotional and spiritual intensity of the pilgrimage, can lead to reduced situational awareness, crowd surges, and delayed response to emergencies. As a result, the Hajj is particularly susceptible to a range of public health and disaster scenarios—including stampedes, heat-related illnesses, disease outbreaks, and incidents. Historical events such as the 2015 Mina stampede, which claimed over 2000 lives, underscore the potential severity of such risks [5]. Given the scale and complexity of the pilgrimage, developing robust, culturally informed disaster preparedness and response strategies is essential to protect both pilgrims and healthcare providers [6].

Further compounding these challenges are evolving global threats such as climate change and pandemics, which intensify the scale and unpredictability of disasters. Rising temperatures increase the risk of heat-related illnesses during mass gatherings, while global pandemics like COVID-19 pose unprecedented challenges for infection control, crowd management, and healthcare system resilience. As such, disaster preparedness now demands not only logistical readiness but also flexibility to address emerging risks [6].

In response to these growing complexities addressing disaster preparedness on a global scale requires coordinated efforts at all levels of governance to ensure effective emergency response. Moreover, local response teams must be well-equipped with resources and expertise, while national frameworks should incorporate prevention, response, and recovery strategies. In this context, healthcare professionals—including RNs, physicians, and paramedics—play a crucial role in disaster response, particularly in mass gathering settings [7]. These professionals provide rapid patient transport, triage, and critical medical interventions, such as airway management, medication administration, and infection control [8]. Furthermore, their collaboration with interdisciplinary teams enhances care planning, communication, and patient advocacy, ensuring a comprehensive and efficient disaster response. To maintain high standards of emergency care, continuous professional development is crucial, as it keeps healthcare professionals updated on evidence-based practices, ultimately leading to better patient outcomes [9].

Despite the critical role of healthcare professionals in disaster preparedness, significant gaps in their readiness and response capabilities persist [10]. The research suggests that many frontline responders receive insufficient training to manage large-scale emergencies, leaving them unprepared for mass casualty incidents [11]. In the United States, for example, studies indicate that a considerable number of healthcare professionals lack the necessary skills to respond effectively to disasters, leading to challenges in triage, resource allocation, and emergency coordination [12]. This lack of preparedness has serious consequences, including delayed response times, increased mortality rates, and overwhelmed healthcare systems [13]. Addressing these deficiencies through structured disaster education, hands-on training, and competency-based learning is essential for improving healthcare professionals’ ability to respond efficiently in emergency situations [14]. Strengthening disaster preparedness frameworks on institutional, national, and international levels is key to ensuring a coordinated and effective response to large-scale disasters [15].

Within the context of the Hajj pilgrimage, disaster preparedness presents even greater challenges, given the unique combination of mass gatherings, extreme weather conditions, and the potential for multiple concurrent emergencies. These factors highlight the critical need for a highly coordinated and efficient disaster response strategy, with RNs, paramedics, physicians, and practice educators playing a pivotal role in emergency preparedness and patient care. Despite the significance of their roles, research on healthcare professionals’ disaster readiness during Hajj remains limited. However, a growing body of literature has begun to explore this domain. Alzahrani and Kyratsis (2017) found that emergency room nurses often lacked sufficient knowledge of emergency and disaster protocols, with over half never having reviewed the disaster response plan and nearly 10% unaware of its existence [16]. Al-Otaibi (2018) further demonstrated that EMS professionals aged 35–39 years and those with Master’s degrees showed the highest levels of disaster preparedness, with paramedics excelling in general readiness and physicians demonstrating a superior understanding of Hajj-specific scenarios [17].

Additional studies further reinforce these trends. Al Wathinani (2018) confirmed that older, highly educated, and military-affiliated EMS personnel exhibited stronger preparedness, particularly when exposed to frequent workshops and hands-on training, underscoring the value of experiential learning [18]. More recently, Attar (2022) reported that healthcare staff in Makkah displayed significantly higher levels of disaster awareness and participation in training drills compared to their counterparts in Madinah and Al Ansar, though gaps in plan familiarity and training coverage were still noted outside Makkah [19]. Together, these studies offer important insights into the demographic and experiential factors influencing healthcare workers’ disaster preparedness. Yet, comprehensive qualitative accounts of frontline providers’ real-time experiences, ethical decision-making, and practical response during Hajj-related disasters remain scarce. Therefore, this systematic literature review aims to assess the existing literature on the disaster preparedness and response experiences of registered nurses and other healthcare professionals within the context of the Hajj pilgrimage in Saudi Arabia.

### Research Questions

1. What is the preparedness of RNs and other HCPs responding to disasters during the Hajj period?
2. What are the experiences of RNs and other HCPs responding to disasters during the Hajj period?
3. What are the views of RNs and other HCPs regarding their disaster education and training during the Hajj?

## 2. Materials and Methods

This section provides a detailed explanation of the process used to conduct the review in accordance with the PRISMA 2020 (Preferred Reporting Items for Systematic Reviews and Meta-Analyses) reporting guidelines [20].

### 2.1. Formulating the Research Questions

The population, intervention, context, and outcomes (PICO) paradigm was used to develop the study’s research questions (Table 1). This framework is appropriate for the review’s research questions and aids in refining the search strategy by highlighting the underlying concepts that must be present in the articles for the question to be answered [21].

### 2.2. Eligibility Criteria

This review included all study designs—quantitative, qualitative, and mixed methods—that were relevant to the aim and research questions, specifically focusing on studies involving healthcare professionals (such as RNs, paramedics, physicians, and practice educators) in relation to their preparedness and experiences during disasters in the Hajj period. Only studies published in English between 2012 and 2025 were included to ensure coverage of the most recent and relevant literature. Studies were excluded if they involved nursing students who had limited clinical responsibilities, did not focus specifically on disasters and healthcare professionals, were not published in English, or consisted of unpublished literature, dissertations, abstracts, or conference proceedings.

### 2.3. Search Strategy

#### 2.3.1. Database

To ensure that as many publications as possible were found that might be relevant to the research questions, the reviewers used alternative terms for the elements of the PICO questions. In addition, the Boolean operators ‘AND’ and ‘OR’ were utilized in the search process to mix different keywords or phrase combinations. Wildcard searching, or truncation, was also implemented so that searches may encompass variations in word endings; for instance, ‘nurse*’ might yield ‘nurses’ and ‘nursing’ rather than just ‘nurse’. The search method was conducted at the QUB library that provides comprehensive access to varied databases for searching and identifying previously published research relevant to the aims of the literature review. These databases provide sophisticated search tools that can help users quickly capture specific pieces of information they require. PubMed, Medline, CINAHL, APA PsycINFO, and Embase were the databases used by the researchers (see Table 2).

#### 2.3.2. Study Screening

**Figure 1:**
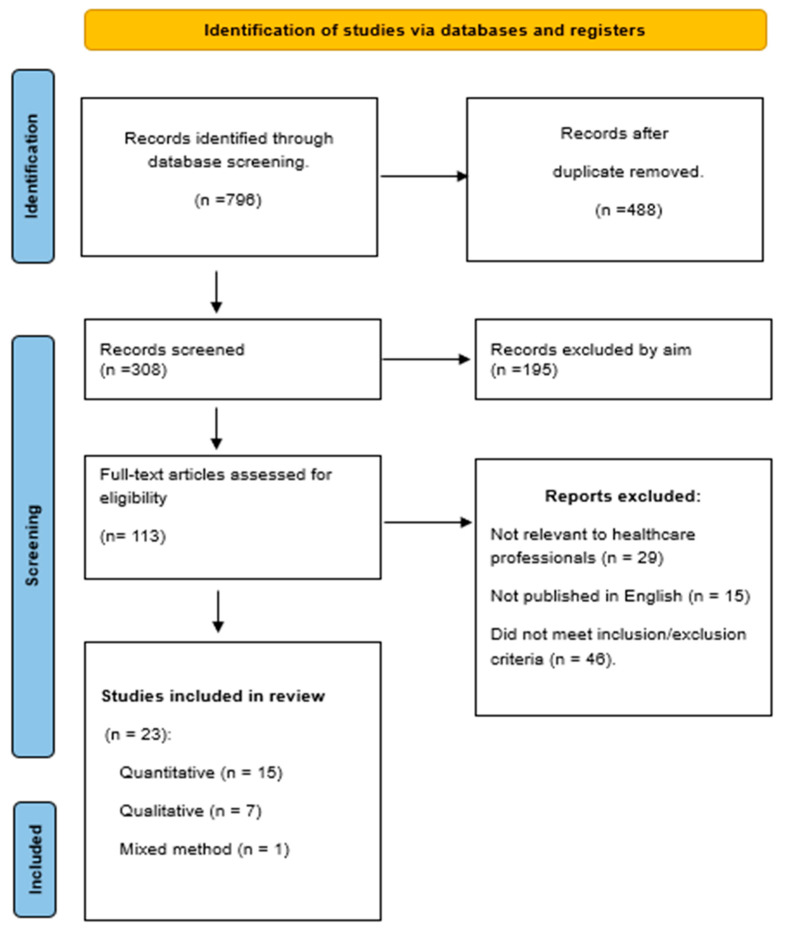
PRISMA flow chart of the search strategy and outcomes.

A total of 796 studies were initially identified through database searches. After the removal of duplicates, 308 records remained for screening. Two reviewers independently screened the titles and abstracts based on predefined eligibility criteria, resulting in the exclusion of 195 studies that did not meet the review’s aim. The full texts of the remaining 113 articles were then independently assessed by the same two reviewers (T.A. and M.B.), with any disagreements resolved through consultation with two additional reviewers (B.R. and L.M.). Following the full-text review, 23 studies were identified as meeting the inclusion criteria and were included in the final synthesis. The study selection process is summarized in the PRISMA flow diagram in Figure 1.

### 2.4. Quality Appraisal

To assess the quality of the included studies, all were subject to appraisal using the Mixed Methods Approval Tool [22]. The MMAT is a five-question quiz with a yes/no/can’t tell option. The MMAT classifies research strategies as either qualitative, quantitative, non-randomized, descriptive, or mixed study. The five questions assist the reviewer to assess the overall research quality of the individual studies. As an integral part of the quality appraisal process, the study supervisors reviewed the included studies.

### 2.5. The Extracted Data

The table below is a summary of the extracted data from the included studies, showing key study characteristics such as the author(s), year of publication, aim, participants, methods, results, and recommendations (Table 3).

### 2.6. Data Analysis

The review used narrative analysis to identify themes aligned with the aims and research questions of the study [21]. Through this systematic approach, the data were examined to detect recurring patterns, leading to the development of clearly defined main themes and sub-themes.

## 3. Results

### 3.1. Search Outcome

A total of 23 studies met the inclusion criteria and were included in the final review. These included studies by Hutton et al. [23], Al-Romaihi et al. [24], Attar [19], Brinjee et al. [25], Al Khalaileh [26], Setyawati et al. [27], Kawasaki et al. [28], Feizolahzadeh et al. [29], Sangkala et al. [30], Al-Otaibi [17], Al Wathinani, A. [18], Naser and Saleem [31], Alzahrani and Kyratsis [16], VanDevanter et al. [32], Alshehri [33], Labrague et al. [34], Al Thobaity [35], Wenji et al. [36], Aliakbari et al. [37], Aliakbari et al. [38], Arbon et al. [39], Ranse [40], and Al Khalaileh et al. [41].

### 3.2. Study Design, Quality, and Appriasl

A total of 15 of the 23 studies utilized the quantitative research method of ‘descriptive survey design’ [16,17,18,19,21,24,25,26,27,30,31,34,35,36,41]. Seven studies conducted the qualitative research method of ‘semi-structured interview’ [23,28,29,37,38,39,40]. One study used the mixed method research of ‘descriptive survey design, semi-structured interviews, and focused group’ [33].

Using the Mixed Methods Appraisal Tool (MMAT), 13 studies achieved a 100% quality score, meeting all five criteria. Another seven studies scored 75%, indicating that most criteria were met but with minor limitations. The reasons for the 75% score included limited detail on the interpretation of results based on data analysis and a lack of coherence between data sources, collection methods, and analysis. Three studies received a 50% score due to the absence of a clearly identifiable research question and inadequate alignment between the research question and data collection methods.

### 3.3. Overview of the Included Studies

The included studies reflected research from several geographical locations, including (i) Middle East countries, such as Saudi Arabia, Iran, Jordan, and Yemon [16,17,18,19,21,25,26,29,31,34,36,38,39]; (ii) Eastern and southern Asian countries, including Indonesia, Philippine, China, and Japan [26,27,30,35,37]; (iii) and other countries, such as USA and Australia [23,33,40,41]. In total, 5026 participants were sampled across the 23 studies, with a range of 71–1724 participants for the quantitative studies and 8–55 participants for the qualitative studies.

### 3.4. Narrative Result Synthesis

Given the heterogeneity of the included studies, a narrative synthesis approach was adopted for this review [21]. Narrative synthesis is defined as ‘an approach to the systematic review and synthesis of findings from multiple studies that relies primarily on the use of words and text to summarize and explain the findings of the synthesis’. The results of the included studies are presented using thematic synthesis, which integrates the analysis and conclusions from individual studies to develop overarching themes. Through this process, three key themes were identified (see Table 4).

#### 3.4.1. Disaster Preparedness

The theme revealed three main subthemes: disaster management in mass gatherings, disaster preparedness in routine situations, and awareness of disaster policies and protocols. Together, these subthemes expose key weaknesses in healthcare professionals’ competencies and systemic readiness, which hinder effective disaster response.

1. Mass Gatherings and Hajj Disaster Preparedness

The findings from the studies [16,17,18,19,23,24] across Australia, Qatar, and Saudi Arabia revealed critical insights into disaster preparedness during mass gatherings (MGs), particularly the Hajj pilgrimage. A consistent pattern emerges: preparedness levels vary widely across stakeholders, regions, and professional roles. While some healthcare professionals and emergency responders demonstrate high readiness—especially in regions like Makkah and within military sectors—many others face significant knowledge gaps in chemical, radiation, and biological disasters, insufficient training, and a lack of familiarity with disaster plans. The studies showed that disparities in age, education, job role, and sector affiliation influenced in disaster preparedness and management. Paramedics tend to be more proficient in general preparedness, while physicians excel in event-specific scenarios like Hajj mass gatherings. The studies also highlighted systemic challenges, including inconsistent hospital readiness, poor communication among stakeholders, limited access to standardized protocols, and infrequent hands-on training.

Importantly, stakeholder misalignment—where event organizers, responders, and attendees prioritize different aspects of safety—further complicates effective disaster management. The evidence points to an urgent need for regular interdisciplinary training, stronger inter-agency coordination, real-time risk communication systems, and standardized disaster response plans tailored to mass gatherings.

2. Disaster preparedness in non-mass gathering contexts

The findings across the studies [27,30,31,34,35,39,41] revealed that healthcare professionals across diverse healthcare systems are often insufficiently prepared to manage disaster scenarios effectively. Studies highlight major competency gaps in critical areas such as clinical decision-making, risk assessment, communication, teamwork, and emotional resilience. These weaknesses limit nurses’ ability to respond efficiently in high-stress and resource-constrained disaster environments.

There is also a lack of specific training for managing biological and chemical threats, handling PTSD, and coordinating logistics during large-scale events. Ethical and legal dilemmas—like resource allocation and end-of-life decisions—further strain response capabilities, especially in the absence of formal education in professional ethics and law.

Preparedness levels were notably higher in military healthcare settings, where structured training, frequent drills, and real disaster experiences significantly improved nurses and healthcare professionals’ readiness. Factors such as age, education, full-time employment, and personal disaster planning were also associated with a greater willingness to respond in crisis situations.

3. Awareness of Disaster Policies and Protocols

The studies’ findings [25,31,34,41] consistently revealed a significant gap in healthcare professionals’ awareness of institutional disaster policies and their designated roles. Many lack understanding of key procedures such as triage, communication protocols, equipment use, and response strategies—particularly during mass gatherings. Compared to physicians, nurses were often less involved in disaster planning and policy formation, leading to underutilization during actual emergencies.

This disconnects results in missed opportunities for frontline contributions and undermines coordinated response efforts. Nurses often perceive their roles as limited to caregiving and education, not recognizing their potential as active disaster responders. The resulting ambiguity around responsibilities can cause delays and inefficiencies when rapid response is most needed

#### 3.4.2. Experiences and Challenges in Disaster Response

The studies [25,32,33,36,38] indicate that nurses face substantial challenges during disaster response, largely due to limited training, lack of practical experience, and systemic barriers. Many enter disaster situations without sufficient preparation in incident management systems, triage protocols, or participation in hands-on drills—leading to reduced confidence and compromised performance. These shortcomings are often exacerbated by the realities of disaster settings, including communication failures, resource scarcity, and poor inter-team coordination.

In high-stress environments such as natural disasters, bioterrorism events, or hospital evacuations, nurses must also navigate ethical dilemmas, psychological strain, and high-stakes decision-making under pressure. Despite these obstacles, registered nurses frequently draw on their adaptability, collaboration skills, and leadership qualities to respond to crises. However, their efforts are often hindered by a lack of institutional support, including clear disaster protocols, robust follow-up systems, and continuity of care planning.

#### 3.4.3. Education and Training

Education and training in disaster care are essential for enhancing the preparedness and response capabilities of healthcare professionals. The studies reviewed highlight two key subthemes: (1) Learning and development needs in disaster care, which focuses on the importance of continuous professional development for both nursing students and clinical nurses, and (2) content of disaster care in nursing curricula, which explores the gaps in disaster education within nursing programs and the need for standardized training.

1. Learning and Development Needs in Disaster Care

The studies [25,26,32,33,36] emphasized the critical role of continuous professional development for both clinical nurses and nursing students in enhancing disaster preparedness. The evidence consistently shows that periodic workshops, disaster simulations, and hands-on training markedly improve readiness for real-world emergencies. Structured training is especially beneficial for less experienced nurses, helping to build their confidence and practical competence. Furthermore, the studies highlight the importance of ensuring that nursing educators themselves receive disaster management training, enabling them to effectively prepare future nurses. Collaborative knowledge-sharing—particularly in high-risk or disaster-prone areas—emerges as a key strategy for strengthening preparedness at both the individual and institutional levels.

2. Content of Disaster Care in Nursing Curricula

The studies [26,28,40] highlighted significant gaps in disaster education within formal nursing programs. Disaster preparedness is often inconsistently integrated into curricula, frequently treated as an elective rather than a core component of nursing education. When included, the content is typically limited in both depth and breadth—particularly in specialized areas such as radiological disaster response. Compounding this issue, many nursing educators lack formal training in disaster management and have limited access to appropriate teaching resources. This undermines their ability to effectively prepare students for real-world emergencies. As a result, nursing graduates exhibit uneven levels of disaster readiness, leaving many underprepared to respond competently in crisis situations.

## 4. Discussion

The findings from this systematic literature review revealed that disaster preparedness in healthcare is deeply influenced by contextual factors, with mass gathering events such as the Hajj presenting distinct challenges compared to routine healthcare settings. While a lack of preparedness exists in both environments, their nature—and the cultural, logistical, and operational implications—differ significantly. The findings of this review underscore how the Hajj context amplifies these challenges, revealing the need for tailored strategies that reflect its unique demands on healthcare delivery.

One of the most significant differences lies in the contextual complexity of mass gatherings. The Hajj represents one of the largest annual mass gatherings globally, drawing millions of pilgrims from over 180 countries. This immense diversity introduces not only logistical and clinical challenges but also profound cultural and communicative demands. The findings from the reviewed studies [17,19,35] indicated that HCPs—particularly those stationed in Makkah or affiliated with military systems—tended to demonstrate slight levels of disaster readiness. This was attributed to their exposure to high-volume patient surges and their participation in more frequent disaster drills. However, the preparedness of many professionals outside these specialized environments was inconsistent, with reported deficits in knowledge, protocol awareness, and hands-on training.

These results align with those of previous studies [42,43], which found that nurses working during the Hajj received standardized disaster response training but often experienced confusion regarding their roles during emergencies. Similarly, other studies [44,45,46] emphasized that managing mass gatherings like Hajj requires systems capable of handling rapid patient flow, ensuring cross-agency coordination, and providing culturally sensitive care—all of which remain areas of insufficient preparedness. The present review echoes these concerns, revealing that, although disaster protocols exist, they are often poorly communicated, particularly to nursing and healthcare professionals’ staff. This lack of communication contributes to inefficiencies in emergency response. While nurses may receive training, they often remain underprepared for real-world disaster scenarios [47].

Many healthcare professionals, despite being frontline responders, are underutilized due to their limited involvement in disaster planning and decision-making [23,24,34]. The studies reviewed indicate that nurses often perceive their roles as secondary to physicians or administrators, leading to uncertainty during critical response periods. This confusion, particularly in high-pressure, time-sensitive environments like Hajj, undermines team coordination and delays care delivery [43,47]. The research by Labrague et al. [34] noted that role ambiguity significantly reduces nurses’ self-efficacy in disaster settings. Without a clear understanding of their responsibilities, HCPs may duplicate efforts, overlook key tasks, or wait for direction in moments that demand autonomy.

Addressing the issue of role ambiguity in disaster response requires proactive and system-wide institutional planning. Roles and responsibilities must be clearly outlined in disaster protocols, but more importantly, these must be effectively communicated and reinforced through routine interdisciplinary training. Merely documenting responsibilities is insufficient in high-pressure disaster scenarios—staff must internalize their roles through active engagement [48,49]. Interdisciplinary simulation drills serve as a practical mechanism for operationalizing these roles under realistic conditions. The research by Veenema et al. [45] suggests that such simulations not only improve technical coordination but also strengthen team cohesion and professional confidence, particularly among nurses who often feel sidelined during disaster events. Incorporating nurses and allied health professionals into the policy development process also fosters a sense of ownership, which in turn enhances preparedness and responsiveness during actual emergencies. Al Thobaity et al. [35] emphasized that inclusive planning ensures protocols are grounded in frontline realities, not just administrative ideals.

However, while these solutions show promise, their effectiveness depends heavily on how they are implemented within institutional culture. In some settings, simulation drills are conducted as one-off events with minimal follow-up or reflection, reducing their potential impact. Moreover, the tokenistic inclusion of nurses in planning—without meaningful participation or decision-making authority—risks reinforcing existing hierarchies rather than dismantling them [16,25]. There is also the challenge of sustainability: without regular updates, refresher training, and strong leadership support, role clarity can erode over time. Therefore, although simulation training and inclusive planning are valuable tools, they must be embedded in a broader institutional commitment to collaborative governance, continuous professional development, and accountability. Only then can these strategies lead to lasting improvements in disaster response readiness, especially in culturally complex and high-risk environments like the Hajj [33,34,40].

### 4.1. Strengths and Limitations of the Review

The present review confirmed the trustworthiness of the results through varied measures. First, the review set an explicit research question and aims, and a review of the past literature identifying any knowledge gaps in nurses’ disaster preparedness. Inclusion and exclusion criteria were developed, and relevant databases and keywords were utilized to identify the most relevant literature [18]. Twenty-three articles were identified, and all were evaluated using the MMAT to determine any potential bias. This review did not include less rigorous sources, for instance, the grey literature, expert opinions, case studies, or editorials, as they provide the lowest forms of evidence [48]. All stages of the systematic review were completed carefully and systematically, with regular feedback from the other three researchers (MB, BJ, and LM).

Throughout the review, it was observed that 13 quantitative studies employed different instruments to assess healthcare professionals’ disaster preparedness, with considerable variation in content, structure, and focus (see Table 3). These inconsistencies limit the ability to accurately measure and compare healthcare professionals’ knowledge, skills, and attitudes related to disaster readiness. While some tools emphasized theoretical knowledge, others assessed practical competencies or psychological preparedness, resulting in fragmented evaluations and leaving key dimensions underexplored. Consequently, the instruments used were not entirely sufficient to capture the full scope of nurses’ disaster-related competencies. To address this issue, future research should adopt standardized and validated tools—such as the Emergency Preparedness Information Questionnaire (EPIQ)—which are specifically designed to assess healthcare professionals’ disaster preparedness across multiple domains. The use of such tools would support more comprehensive, reliable, and comparable assessments, ultimately contributing to improved disaster training and readiness among healthcare professionals.

### 4.2. Future Research

This review set out to explore how prepared healthcare professionals—particularly registered nurses and paramedics—are to respond to disasters during the Hajj pilgrimage in Saudi Arabia. Out of the seven studies that met the inclusion criteria [16,17,18,19,25,33,35], only four directly examined experiences during the Hajj itself. The remaining studies looked more broadly at disaster preparedness in general healthcare settings. All used quantitative methods and mostly involved nurses and paramedics only.

Additionally, while the review includes a range of valuable international studies, many come from contexts that differ significantly from the Hajj. As a result, some findings may not fully apply, especially when considering the cultural, religious, and linguistic factors that define healthcare delivery during the pilgrimage. Elements like gender-separated care, multilingual communication needs, and faith-based health practices are often overlooked in the broader literature.

To better understand disaster response in this unique setting, future studies should use qualitative approaches to explore healthcare workers’ real-world experiences. These methods are better suited to uncover the challenges and dynamics professionals face on the ground, including ethical dilemmas, communication barriers, and coordination across teams. It would also be beneficial to include a wider mix of participants—such as physicians, EMS staff, educators, and administrators—to reflect the full scope of disaster response. Research that is rooted in the specific context of the Hajj can help shape culturally appropriate training, improve preparedness, and support more effective disaster response strategies.

## 5. Conclusions

This systematic review identified lack in disaster preparedness, response, and education among registered nurses and healthcare professionals, particularly within the context of the Hajj. The findings highlight deficiencies in hands-on experience, cultural competence, and knowledge of disaster policies, as well as inconsistencies in disaster-related content within nursing education. These limitations affect the ability of healthcare professionals to respond efficiently and ethically in complex, high-risk scenarios such as those presented during mass gatherings. To address these challenges, disaster education programs should integrate a standardized yet adaptable disaster preparedness module that includes content on ethical decision-making, culturally sensitive care, and communication in multilingual environments. This could take the form of a model curriculum co-developed by universities, healthcare institutions, and national agencies such as the Saudi Ministry of Health. Implementation could be supported through institutional partnerships with hospitals serving Hajj regions, where students and healthcare professionals could participate in annual, interdisciplinary simulation exercises modeled on Hajj-specific scenarios.

## Figures and Tables

**Table 1:** PICO paradigm.

Research Questions	Population	Intervention	Context	Outcome
First question	RNs and HCPs	Responding to disasters	Hajj	Preparedness
Second question	RNs and HCPs	Responding to disasters	Hajj	Experiences
Third question	RNs and HCPs	Disaster nursing education and training	Hajj	Learning needs

**Table 2:** Database search results in figures.

Database	Search Terms	Results
CINAHL	“Nursing disaster” OR “Disaster preparedness” OR “Disaster response” AND “Emergency response” OR “Disaster insights”	296
Scopus	“Nursing disaster” OR “Disaster preparedness” OR “Disaster response” AND “Emergency response” OR “Disaster insights”	87
Medline	“Nursing disaster”” OR “Disaster preparedness” OR “Disaster response” AND “Emergency response” OR “Disaster insights”	157
Embase	“Nursing disaster” OR “Disaster preparedness” OR “Disaster response” AND “Emergency response” OR “Disaster experiences”	197
APA PsycINFO	“Nursing disaster” OR “Disaster preparedness” OR “Disaster response” AND “Emergency response” OR “Disaster experiences”	59

**Table 3:** Extracted data.

Citation and Country	Aims	Participants	Methods	Key Findings	Recommendations
Alzahrani and Kyratsis (2017) [16], Saudi Arabia	Assessing the knowledge, roles, and skills of ER nurses for disaster response to mass gathering during the Hajj.	RNs (*n* = 210)	Quantitative, descriptive survey.	ER nurses lack emergency and disaster preparedness knowledge. Over half of ER had not reviewed the plan’s strategy, and nearly 10% did not realize it existed.	This study did not compare hospital training and exercises across four hospitals. This may explain the apparent difference in disaster education, training and exercise participation among study participants and needs further research.
Al-Otaibi, a. (2018) [17], Saudi Arabia	To assess the disaster preparedness knowledge of EMS providers during the 2016 Hajj and examined the influence of demographic factors, training, and knowledge sources on their preparedness.	Paramedics (*n* = 1650)	Quantitative, descriptive survey design.	The study found that EMS providers aged 35–39 years and those holding Master’s degrees demonstrated the highest levels of disaster preparedness knowledge. Paramedics had stronger general preparedness, while physicians showed better understanding of Hajj-specific scenarios. Providers from the military sector were more knowledgeable compared to those in civilian sectors. Additionally, frequent and longer workshops, as well as real disaster experience, were key factors in improving preparedness levels.	Increase training frequency and duration, emphasizing hands-on workshops and drills. Enhance continuing education and university-based disaster content. Revise EMS scope of practice to better align with mass gathering needs like the Hajj.
Al Wathinani, A. (2018) [18], Saudi Arabia	Assessing the disaster preparedness knowledge of Saudi Red Crescent Emergency Medical Service professionals during the Hajj 2016 mass gathering.	Paramedics (*n* = 1650)	Quantitative, descriptive survey design.	The study found that older, highly educated, and military sector EMS professionals had better disaster preparedness knowledge, with paramedics excelling in general preparedness and physicians in Hajj-specific knowledge. Frequent workshops (4 times/year) and hands-on training significantly improved preparedness, with real disaster experiences and continuing education being key knowledge sources.	The study highlights the need for structured training programs and frequent drills to enhance emergency response during the Hajj.
Attar, A. (2022) [19], Saudi Arabia	To assess and compare disaster preparedness knowledge and attitudes among healthcare workers in the holy mosques of Makkah and Madinah.	Healthcare professionals (*n* = 314)	Quantitative, descriptive survey.	Makkah Health Center workers showed significantly higher preparedness levels, awareness of disaster plans, and involvement in training and drills than those in Madinah and Al Ansar. Most staff were willing to work during crises, but gaps existed in plan familiarity and training coverage, especially outside Makkah.	Enhance disaster training and drills in Madinah and Al Ansar facilities. Promote comprehensive awareness of disaster plans across all roles. Strengthen resources and build confidence among staff to ensure safety and readiness during mass gatherings like the Hajj and Umrah.
Hutton et al. (2025) [23], Australia	To explore safety at mass gathering (MG) events through the perspectives of event organizers, emergency responders, and attendees.	Healthcare professionals (*n* = 15)	Qualitative, interviews.	Different safety priorities among stakeholders: Event organizers focus on crowd control and logistics. Emergency responders prioritize medical preparedness. Attendees’ emphasize personal safety and security. Need for improved multi-stakeholder collaboration. Effective communication and real-time risk management are crucial for MG safety.	The findings emphasize that building trust and improving coordination among stakeholders are crucial for ensuring safety at MG events.
Al-Romaihi et al. (2023) [24], Qatar	To assess the knowledge, attitude, and training of healthcare workers and the preparedness of hospital emergency departments for communicable disease threats during mass gathering (MG) events.	Physicians and RN (*n* = 783)	Quantitative, descriptive survey.	Many healthcare workers had basic knowledge, but gaps existed in infection control measures and response protocols. Variability in hospital readiness, with some lacking standardized response plans. Preparedness differed between hospitals, affecting the ability to handle MG-related outbreaks.	The findings emphasize the need for regular drills and updated training programs to enhance emergency response readiness.
Brinjee et al. (2021) [25], Saudi Arabia	Identifying the most essential education and training for ER nurses in Taif city to prepare for catastrophes.	RNs (*n* = 210)	Quantitative, descriptive survey.	RNs with fewer than three years of experience lack expertise of incident management systems, disaster triage, and disaster drills.	Greater emphasis should be placed on nursing education and training in disaster management, while hospitals should conduct more drills centered on disaster response to enhance nurses’ knowledge and skills in handling emergency situations.
Al Khalaileh et al. (2021) [26], Jordan	Exploring nurse educators’ views on disaster preparedness curriculum inclusion.	Nursing educators (*n* = 131)	Quantitative, descriptive survey.	Most nursing educators had no disaster management training or access to disaster management literature. Participants mphasizes the importance of disaster management training for nursing educators to better inform their students.	More research needs to be conducted to find out what problems and opportunities there are for embedding/including disaster management into the nursing curriculum.
Setyawati et al. (2020) [27], Bengkulu, Indonesia	This study aimed to determine the factors affecting the disaster preparedness of RNs in Bengkulu, Indonesia.	RNs (*n* = 130)	Quantitative, descriptive correlational study.	RNs in Bengkulu reported a moderate level of disaster knowledge, skills, and preparedness. Disaster knowledge and skills were significantly correlated with preparedness, with key influencing factors being educational level, disaster knowledge, and disaster skills.	Qualitative study on RNs’ disaster management experiences, obstacles, and learning requirements may help future researchers build successful training and practice interventions.
Kawasaki et al. (2020) [28], Japan	The study aimed to determine the essential radiological education needed for public health nurses based on their experiences during the Fukushima nuclear disaster.	PHNs (*n* = 8)	Qualitative, interviews.	The study found that many PHNs lacked adequate radiation knowledge, making it difficult for them to support evacuees and manage shelters effectively. Risk communication training was deemed crucial in helping PHNs address public concerns and provide accurate information during nuclear disasters.	It was advised in the study to incorporate the radiation disaster into nursing education.
Feizolahzadeh et al. (2019) [29], Iran	Identifying barriers and facilitators to disaster patient continuity.	Health professionals (*n* = 24)	Qualitative, interviews.	The study identified seven key barriers to continuity of care for dischargeable patients in disasters, including lack of disaster paradigm, poor coordination, inadequate hospital preparedness, and weak pre-hospital systems. Key facilitators included establishing patient follow-up systems, improving hospital disaster preparedness, and integrating disaster management training into medical education.	Addressing these barriers and implementing structured policies can enhance patient care continuity and hospital surge capacity during disasters.
Sangkala et al. (2018) [30], South Sulawesi Indonesia	This study examines CHN coordinators’ perceptions of disaster preparedness and learning needs for managing natural disasters in community health settings in South Sulawesi, Indonesia.	RNs (*n* = 214)	Quantitative, descriptive survey design.	The study revealed that RNs were unprepared to help their communities. RNs could not design or promote state and federal guidelines and crisis plans. Nurses were unprepared for crises in PTSD, mental counselling, and biohazard control.	Future studies should interview nurses, disaster team members, nursing educators, disaster managers, and policymakers to assess disaster management and determine learning requirements. Action research or experimental studies may be needed to evaluate educational programs based on these findings.
Naser and Saleem (2018) [31], Yemen	Assessing the health professionals’ disaster preparedness knowledge, attitudes, and training.	Health professionals (*n* = 531)	Quantitative, cross-sectional survey.	The study found that Yemeni health professionals had insufficient knowledge of disaster preparedness, with only 32% having good knowledge, while 41% had never received training, and 58.9% had not participated in emergency exercises. Postgraduate staff and physicians were more knowledgeable, but health administrators lacked adequate training.	The study recommends integrating disaster preparedness into medical curricula, offering long-term training, and conducting multi-agency simulations to improve disaster response.
VanDevanter et al. (2017) [32], USA	Exploring nurses’ perspectives: what were their problems and resources, and how did Hurricane Sandy affect nursing education and disaster preparedness?	RNs (*n* = 189)	Mixed methods (survey and interviews).	The study found that nurses faced significant challenges during the Hurricane Sandy hospital evacuation, including limited disaster training, communication breakdowns, and resource shortages. Nurses relied on teamwork, leadership, and adaptability to manage the crisis, despite a lack of formal preparedness.	The study highlights the need for enhanced disaster training, hands-on simulations, and clear policies to improve nurses’ disaster response capabilities.
Alshehri (2017) [33], Saudi Arabia	Determination of disaster preparedness for ED nurses.	RNs (*n* = 72)	Quantitative, descriptive survey.	The study highlighted that respondents had minimal disaster experience, reflected in their low confidence levels post-disaster involvement.	The study underscores the necessity for ongoing efforts to enhance disaster training and ensure nurses are adequately prepared
Labrague et al. (2016) [34], Philippines	Investigating the level of preparedness of nurses in the case of an emergency.	RNs (*n* = 170)	Quantitative, descriptive survey.	The study found that 80% of nurses in the Philippines felt inadequately prepared for disasters, with 57.7% unaware of disaster management protocols in their workplaces. Nurses identified their roles mainly as educators, caregivers, and counsellors, but participation in disaster training programs was low.	The study highlights the need for improved disaster education, protocol implementation, and integration of preparedness training into nursing curricula.
Al Thobaity (2015) [35], Saudi Arabia	Identifying disaster management knowledge among military and civilian nurses.	RNs (*n* = 384)	Quantitative, descriptive survey.	The findings revealed that nurses possessed moderate knowledge of disaster preparedness, with military hospital nurses demonstrating higher levels of knowledge compared to their counterparts in government hospitals. Notably, most nurses acquired their disaster management knowledge and skills primarily through participation in disaster drills.	The study underscores the necessity for enhanced education in all facets of disaster management for nurses, emphasizing the importance of clearly defining their roles during disaster response.
Wenji et al. (2015) [36], China	Providing an overview of the experiences gained by Chinese nurses who assisted with relief efforts following the earthquakes in Wenchuan and Yushu.	RNs (*n* = 12)	Qualitative, interviews.	The study found that Chinese nurses faced significant challenges during earthquake relief efforts, including poor living conditions, lack of medical resources, and psychological stress. Key issues identified were inadequate disaster planning, the need for stronger teamwork and critical thinking skills, and insufficient disaster education.	The findings highlight the urgent need for specialized disaster nursing training and improved disaster response coordination in China.
Aliakbari et al. (2015) [37], Iran	Exploring the experiences and ethical competencies of Iranian nurses in disaster response.	RNs (*n* = 35)	Qualitative, interviews.	The study found that Iranian nurses faced significant ethical and legal challenges during disaster response, highlighting the importance of professional ethics and adherence to legal standards. Nurses emphasised the need for ethical decision-making skills and awareness of legal responsibilities in crisis situations.	Nursing education should cover catastrophe nursing ethics and law. Nurse educators must realise that ethics and law help students conceptualise circumstances and find ethical and legal answers in disasters. Nurses’ crisis response preparation needs more research.
Aliakbari et al. (2014) [38], Iran	Identifying the technical competencies required for nurses in responding to disasters.	RNs (*n* = 30)	Qualitative interviews.	The study identified key technical competencies for Iranian nurses in disaster response, including clinical skills, risk assessment, decision-making, communication, teamwork, and adaptability. Nurses emphasized the importance of ethical and legal awareness in disaster situations.	The study highlights the need to integrate these competencies into nursing education and training programs to enhance disaster preparedness.
Arbon et al. (2013) [39], Australia	To investigate the disaster participation willingness among staff in four emergency services.	RNs (*n* = 54)	Qualitative (focus group, interviews)	The study found that emergency nurses in Australia were more willing to work during conventional disasters if they had formal disaster education, a family disaster plan, no childcare responsibilities, and worked full-time. It highlights the importance of workplace preparedness and disaster training in increasing nurses’ willingness to respond.	The study recommends enhancing disaster education, workplace preparedness, and support systems to improve nurses’ willingness to respond. Future research should explore barriers to participation in different disasters and strategies to support healthcare workers with family responsibilities.
Ranse (2013) [40], Australia	Exploring the nursing disaster content in Australian postgraduate courses.	Course convenors (*n* = 12)	Quantitative, descriptive survey.	The nursing disaster content in courses are varied both in the types of topics and duration. Most nursing disaster content included: types of disasters, hospital response, nurses’ roles, triage, and management of dying people.	Incorporation of the core competencies such as those from the International Council of Nurses and WHO might enhance content consistency in the curriculum. Create a proposal for disaster health content at national level.
Al Khalaileh et al. (2012) [41], Jordan	Measuring Jordanians’ conceptions of their own disaster management abilities.	RNs (*n* = 474)	Quantitative, descriptive survey.	Nurses lacked disaster planning awareness and skills in implementing and formulating new disaster guidelines and. RNs felt unprepared for biological and chemical attacks, organizational logistics, and group A-B biological weapons.	All undergraduate nursing programs should include disaster management training to better prepare future nurses. More research is needed to understand RNs’ emergency preparedness challenges.

**Table 4:** Result themes.

Theme	Subtheme	Studies
Disaster Preparedness	Mass Gatherings and Hajj Disaster Preparedness	[16,17,18,19,23,24]
	Disaster Preparedness in Non-Mass Gathering Contexts	[27,30,31,34,35,39,41]
	Awareness of Disaster Policies and Protocols	[25,31,34,41]
Experiences and Challenges in Disaster Response	Experiences and Challenges in Disaster Response	[25,32,33,36,38]
Education and Training	Learning and Development Needs in Disaster Care	[25,26,32,33,36]
	Content of Disaster Care in Nursing Curricula	[26,28,40]

## Data Availability

No new data were created or analyzed in this study.
